# Extraction, Chemical Composition and Insecticidal Activities of *Lantana camara* Linn. Leaf Essential Oils against *Tribolium castaneum*, *Lasioderma serricorne* and *Callosobruchus chinensis*

**DOI:** 10.3390/molecules29020344

**Published:** 2024-01-10

**Authors:** Kolapparamban Aisha, Naduvilthara U. Visakh, Berin Pathrose, Nicola Mori, Rowida S. Baeshen, Rady Shawer

**Affiliations:** 1 Department of Agricultural Entomology, College of Agriculture, Kerala Agricultural University, Thrissur 680656, India; 2 Department of Biotechnology, University of Verona, 37114 Verona, Italy; 3 Department of Biology, Faculty of Science, University of Tabuk, Tabuk 71421, Saudi Arabia; 4 Department of Plant Protection, Faculty of Agriculture (Saba Basha), Alexandria University, Alexandria 21531, Egypt

**Keywords:** essential oils, insecticidal activity, phytotoxicity, *Tribolium castaneum*, *Lasioderma serricorne*, *Callosobruchus chinensis*, fumigation toxicity, contact toxicity, repellent activity, *Lantana camara*

## Abstract

Storage pests and the food spoilage they cause are problems of great concern. Using essential oil obtained from different plants as an insecticide against these storage pests can be considered an environmentally friendly pest management option. *Lantana camara* Linn. (family Verbenaceae) is a flowering species, and is also a noxious weed that can proliferate well in nearly all geographical habitats. A biopesticide derived from the essential oil extracted from this plant can offer an effective solution for controlling storage pests. The goal of this study is to extract and analyse the chemical composition of essential oil obtained from *L. camara* leaves, and assess its effectiveness as a bioactive substance against three storage pests: *Tribolium castaneum*, *Lasioderma serricorne*, and *Callosobruchus chinensis*. The yield of essential oil extracted from *L. camara* leaves was about 0.24 ± 0.014%. By employing the GC-MS technique, the major phytochemicals contained in *L. camara* leaf essential oil were identified as caryophyllene (69.96%), isoledene (12%), and ɑ-copaene (4.11%). The essential oil exhibited excellent fumigant toxicity (LC_50_ of 16.70 mg/L air for *T. castaneum*, 4.141 mg/L air for *L. serricorne* and 6.245 mg/L air for *C. chinensis* at 24 h), contact toxicity (LC_50_ of 8.93 mg/cm^2^ for *T. castaneum*, 4.82 mg/cm^2^ for *L. serricorne* and 6.24 mg/cm^2^ for *C. chinensis* after 24 h) along with effective repellent activity towards the test insects. In addition, the oil showed no significant phytotoxicity on the germination of paddy seeds. This presents the potential to utilize a weed in developing a biopesticide for effectively managing stored product insects because of its strong bioactivity.

## 1. Introduction

Plants that have been introduced to new regions for ornamental purposes are one of the largest pools of alien species. Some of them have naturalized and invaded their new regions, posing greater threats to the ecosystem [1]. One such example is *Lantana camara* Linn., also known as wild or red sage (Family: Verbenaceae), an invasive alien species that originated in tropical America. *Lantana camara* has spread to over 60 countries [2] and is one of the world’s top 100 most invasive species and 10 worst weeds as per IUCN [3]. It has a prolific mechanism for seed disposal and is potent enough to disrupt the structure of terrestrial ecosystems [4]. The allelopathic effect of lantana can also contribute to its success in different environmental conditions. It can interfere with the growth and succession of native species, thus reducing biodiversity [3]. It can outgrow agricultural lands with dense thickets, block the passage of livestock, and poison them [4]. Thus, this noxious weed ought to be managed. Employment of a problematic weed, harnessing the potent bioactivity of its secondary metabolites, especially the essential oil, would find significant demand in the present world.

Dried and stored food products such as grains and pulses, processed food materials, other durable agricultural commodities, and non-food derivatives of agricultural products are under significant damage from storage pests worldwide. Storage pests can cause postharvest losses of up to 20% in developing nations and 9% in developed nations. Furthermore, they contaminate the products by adding live and dead insects, body parts, chemical waste, etc. Traditional methods of storage pest control relied upon various physical, chemical, and biological methods by mixing stored grains with various protectants made of plant products like neem and pongamia leaves [5].

These traditional control measures have been replaced by synthetic chemicals, such as fumigants, grain protectants, and disinfectants, due to the quick development of chemical synthesis processes. Chemical insecticides were thought to be the most efficient management tool for a long time. Still, a serious threat to the prolonged use of such insecticides is the development of resistance in the target pest [5]. Chemical insecticides like malathion and chlorpyrifos leave pesticide residues in grains. Fumigants like aluminium phosphide can cause toxic effects through ingestion and inhalation [6]. Therefore, there is a need for safer and more eco-friendly alternatives to replace these synthetic chemicals.

Essential oils are highly pure, secondary metabolites extracted from various plant parts like stems, flowers, leaves, seeds, roots, resins, fruit rinds, etc. Researchers have conducted various studies to discover the different biological activities of essential oils, including insecticidal activities [7,8,9,10]. These are effective alternatives against synthetic chemicals due to their insecticidal properties as larvicidal, ovicidal, antifeedant, repellent, and growth inhibitor [11]. Essential oils are generally considered safe for humans [12]. In the last several years, much research has been performed on the insecticidal effect of different essential oils against storage pests. This research has examined the contact, fumigant, and repellent action of essential oils [11,13].

This research is concerned with three different test insects, *Tribolium castaneum* (Coleoptera: Tenebrionidae: red flour beetle), *Lasioderma serricorne* (Coleoptera: Ptinidae; cigarette beetle), and *Callosobruchus chinensis* (Coleoptera: Chrysomelidae; pulse beetle). *Tribolium castaneum* is the most destructive storage pest with a very broad host range, particularly attacking processed rice and wheat. The population growth rate of *T. castaneum* is one of the highest among all storage pests due to its high reproductive rate and long reproductive life [14]. Cigarette beetle is a cosmopolitan storage pest that causes tremendous economic losses. It can feed on a variety of products, the most common being cured tobacco leaves [15]. Pulse beetle is a major storage pest of all types of pulses. The damage caused by the pest can be as high as 50% in pulses like cowpea, Bengal gram, red gram, and green gram [16]. All three pests cause severe damage to stored products all around the globe and have developed resistance against insecticides. So, an effective management method other than the conventional methods needs to be employed against these storage pests.

Studies on the many different biological properties of *L. camara* essential oil have been carried out, such as anthelminthic activity, anti-protozoal activity, insecticidal activity, anti-viral activity, antioxidant activity, antifeedant activity, phytotoxic activity, antibacterial, antifungal activity, modulatory activity, etc. [17]. Fumigant toxicity bioassay of lantana essential oil against *Sitophilus oryzae* [7], bioassay of lantana oil against *Callasobruchus* sp. [18], the effect of leaf essential oils of goat weed, red sage and siam weed on the mortality of maize weevil [19], etc. are some of the research carried out on the insecticidal activities of lantana essential oil against storage pests. However, not many research studies have been conducted on the repellency, fumigation, and contact toxicities of *L. camara* essential oil against three major storage pests *T. castaneum*, *L. serricorne*, and *C. chinenesis* in depth.

In the above context, the extraction of bioactive essential oils from *L. camara* weed, exhibiting potent insecticidal activities, provides a viable alternative to synthetic pesticides in the realm of sustainable agriculture. This study is focused on the isolation and characterization of oil from lantana leaves and the assessment of its insecticidal activities like repellency, contact toxicity, and fumigant toxicity against three major storage pests *T. castaneum*, *L. serricorne*, and *C. chinenesis*.

## 2. Results

### 2.1. Yield and Chemical Composition Analysis by GC-MS/MS

**Figure 1:**
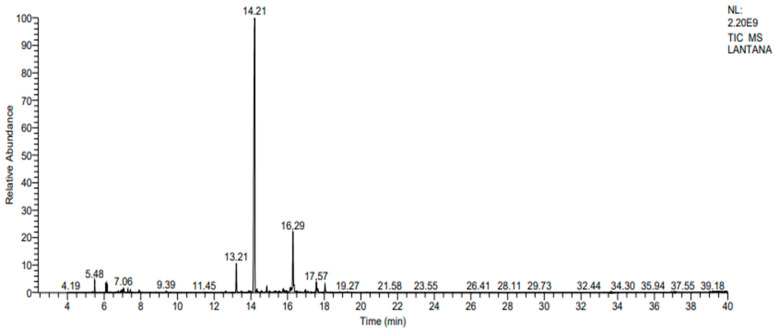
The GC-MS chromatograms of *L. camara* leaf oil.

The leaf of *L. camara* yielded about 0.24 ± 0.01% essential oil by hydrodistillation using the Clevenger apparatus. The GC-MS/MS technique disclosed the presence of 24 different chemical components with a total retention time of 99.21% (Figure 1, Table 1). The major chemical constituents identified were caryophyllene (69.96%), isoledene (12%), and ɑ-copaene (4.11%). It is pertinent to note that the phytochemical caryophyllene makes up nearly 70% of the essential oil.

### 2.2. Contact Toxicity

The contact toxicity bioassay of *L. camara* essential oil indicates that various essential oil concentrations considerably affected all three test insects (Table 2). *Lasioderma serricorne* and *C. chinensis* showed almost 100% mortality at concentrations between 1 mg/cm^2^ and 9 mg/cm^2^, whereas *T. castaneum* took a concentration of up to 12 mg/cm^2^ to reach near 100% mortality. Probit analysis showed that the LC_50_ value of *T. castaneum* against *L. camara* essential oil after 24 h exposure was 8.93 mg/cm^2^, and the LC_90_ value was 13.54 mg/cm^2^. The LC_50_ and LC_90_ values of *T. castaneum* against the essential oil at 48 h exposure were 7.92 mg/cm^2^ and 10.47 mg/cm^2^, respectively. Similarly, the LC_50_ and LC_90_ values of *L. serricorne* against the essential oil after 24 h exposure were 4.82 mg/cm^2^ and 17.47 mg/cm^2^, and after 48 h exposure were 3.41 mg/cm^2^ and 15.57 mg/cm^2^, respectively. Among the three test insects, *C. chinensis* showed the highest susceptibility towards the essential oil with an LC_50_ and LC_90_ of 1.83 mg/cm^2^ and 6.27 mg/cm^2^ at 24 h exposure and 0.45 mg/cm^2^ and 5.06 mg/cm^2^ at 48 h exposure. As the concentration and exposure time increased, the mortality rate (%) rose among all the tested insects. *T. castaneum* showed the least toxicity towards lantana essential oil.

### 2.3. Fumigant Toxicity

*Lantana camara* essential oil significantly affected the test insects by fumigant action. The LC_50_ and LC_90_ of each test insect at 24 h and 48 h exposure were found by probit analysis (Table 3). *L. serricorne* was the most vulnerable towards the fumigant bioassay with LC_50_ and LC_90_ values of 4.14 mg/L air and 10.91 mg/L air, respectively, at 24 h exposure. The LC_50_ and LC_90_ at 48 h exposure were 2.53 mg/L air and 8.62 mg/L air. *Callosobruchus chinensis* showed LC_50_ and LC_90_ values of 6.24 mg/L air and 27.36 mg/L air at 24 h exposure and 3.07 and 25.85 at 48 h exposure. *Tribolium castaneum* showed the least fumigant toxicity when compared with *L. serricorne* and *C. chinensis* with an LC_50_ and LC_90_ of 16.70 mg/L air and 23.21 mg/L air at 24 h exposure and 14.47 mg/L air and 20.97 mg/L air at 48 h exposure.

### 2.4. Repellent Activity

**Figure 2:**
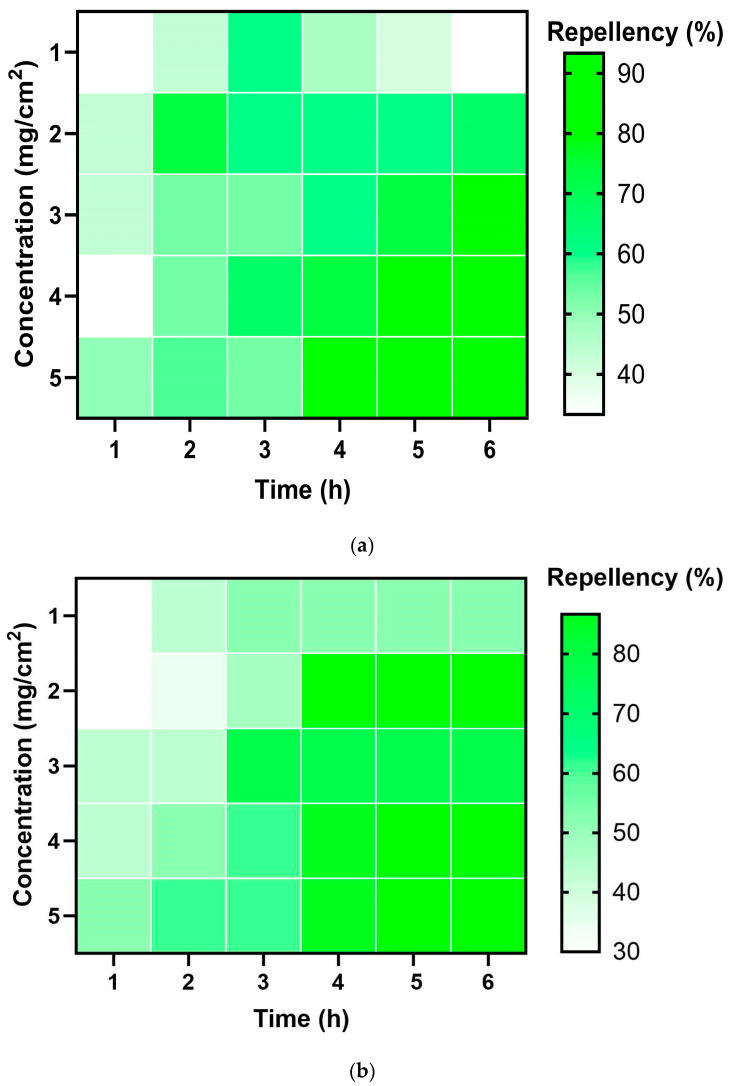

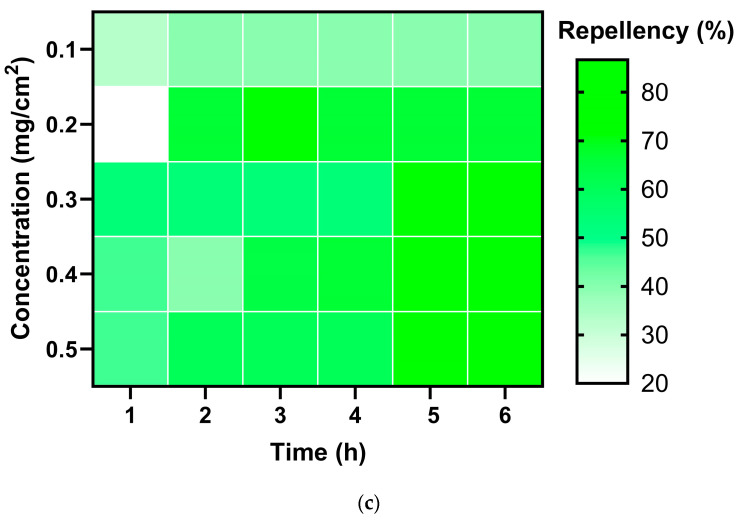
(**a**) Repellency (%) of lantana leaf essential oil against *T. castaneum*; (**b**) repellency (%) of lantana leaf essential oil against *L. serricorne*; and (**c**) repellency (%) of lantana leaf essential oil against *C. chinensis*.

The repellent assay of *L. camara* oil revealed significant repellent activity of the essential oil (Table 4). The mean repellence of test insects varied depending on the concentration and increased with an increase in concentration (Figure 2). *C. chinensis* showed a mean repellence of more than 50% at a concentration above 0.2 mg/cm^2^, and *T. castaneum* and *L. serricorne* showed the same at a concentration above 2 mg/cm^2^. At the lowest concentration of 0.1 mg/cm^2^, *C. chinenesis* showed a mean repellence of 37.7% (class II), which increased drastically to 56.10% (class III) at 0.2 mg/cm^2^. A value of 65.55% (class IV) was the highest mean repellence shown among the three test insects, by *T. castaneum* at a concentration of 5 mg/cm^2^. The highest mean repellence shown by *L. serricorne* and *C. chinensis* was 64.44% (class IV) at a concentration of 5 mg/cm^2^ and 0.5 mg/cm^2^, respectively (Figure 2).

### 2.5. Phytotoxicity Study of Essential Oil on Grains

**Figure 3:**
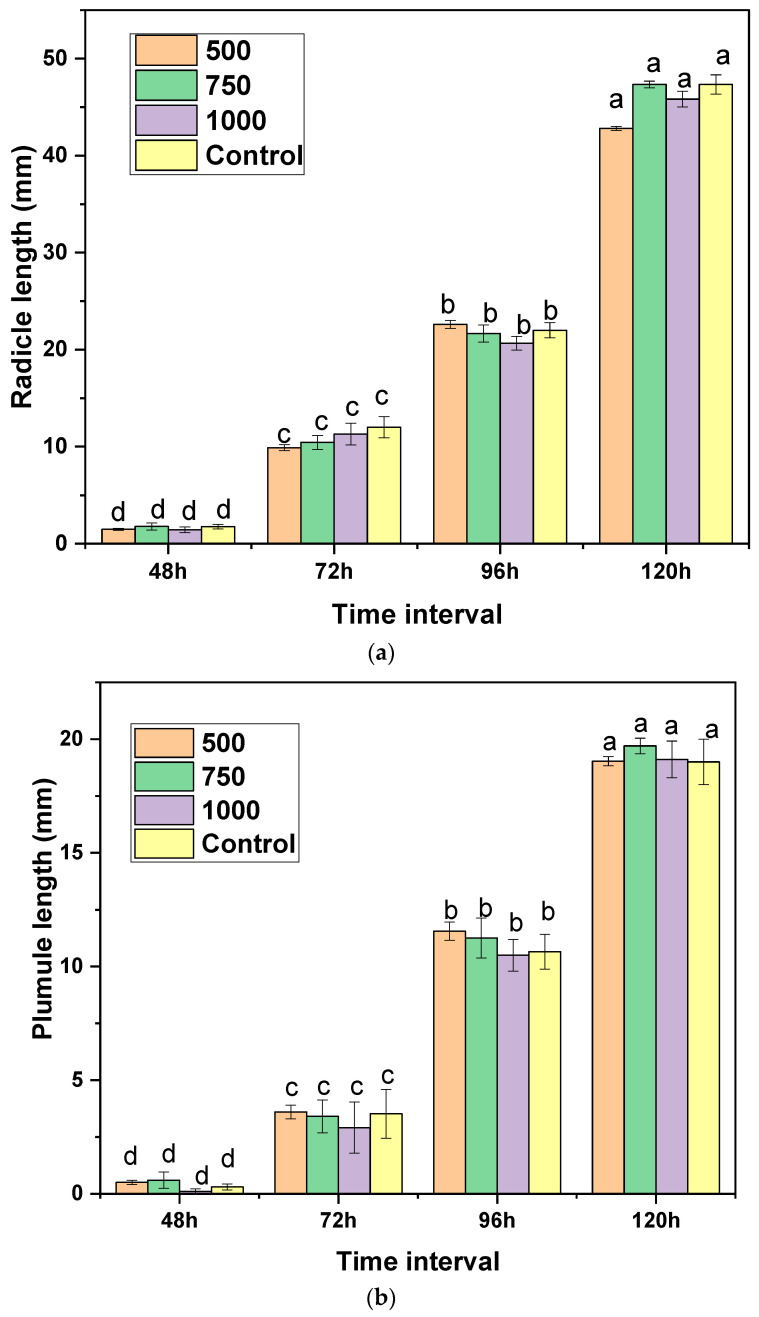
Phytotoxicity check of *L. camara* essential oil on seedling growth of rice: (**a**) radicle; (**b**) plumule (same letters do not differ significantly (*p* < 0.05)).

Any biopesticide applied to stored seeds and other commodities should not impact their growth and germination. To find out the potential phytotoxicity of *L. camara* essential oil, paddy seeds were treated with different concentrations of *L. camara* essential oil. The three different concentrations taken in this study were 500, 750 and 1000 μg/mL, and seeds with no treatment were taken as the control. No significant effect on the germination percentage of seeds could be observed (Table 5). The radicle and plumule length of seedlings did not show any significant phytotoxicity at increasing concentrations (Figure 3a,b). Malformation of the seeds was not observed in all treatments. Hence, it is understood that *L. camara* essential oil is safe to be applied on stored products as it lacks phytotoxicity.

## 3. Discussion

GC-MS/MS analysis revealed that caryophyllene, ɑ-copaene, and isoledene are the major components of lantana essential oil. Many other research studies align with this result, showing that caryophyllene is the major component of *L. camara* essential oil [20,21,22,23,24,25]. *L. camara* was found to have a rich source of monoterpenes and sesquiterpenes, the most common major constituent being the sesquiterpene, α and β caryophyllene [26,27]. However, the chemical components and their percentage composition can vary based on the geographical area, and climatic and soil conditions where the plants were grown. It can also depend on the harvest time and the plant’s growth stage [28]. In research regarding the phytochemical characterization of lantana essential oils from Algeria, the percentage composition of chemical constituents varied based on the month of harvest and over different years, caryophyllene being the major component in all cases [7].

The study showed that *L. camara* essential oil is efficient enough to cause contact toxicity towards all three test insects. The essential oil was more vulnerable to pulse beetle than cigarette beetle and red flour beetle. The LC_50_ value of all test insects decreased with increased exposure time. Various other studies on the contact toxicity of *L. camara* essential oil on other coleopteran storage pests followed the same trends. Numerous studies have shown the significant contact toxicity of *L. camara* against various coleopteran storage pests [19,29,30]. However, the toxicity may vary depending on the test insect and the essential oil constituents. The essential oil derived from *Myristica fragrans* showed high contact toxicity towards cigarette beetles with an LC_50_ value of 19.3 μg/adult while *L. camara* essential oil possessed an LC_50_ of 4.82 mg/cm^2,^ indicating a higher contact toxicity of *L. camara* essential oil than *M. fragrans* [31]. The biological activities of *L. camara* are attributed to constituents like caryophyllene, 1,8-cineole, sabinene, pinene, and others. Moreover, caryophyllene II oxide and aromadendrene II oxide isolated from *L. camara* essential oil were found to have insecticidal activities [32,33]. In an experiment to study the contact toxicity of the sesquiterpene, acetonic solutions of pure β-Caryophyllene were administered against maize weevil, *Sitophilus zeamais* and notable lethality upon contact toxicity was recorded [34]. Upon the insecticidal evaluation of essential oil derived from a piper species native to Africa named *Piper guineense*, remarkable grain contact toxicity and filter paper contact toxicity were recorded, the major components of the essential oil being caryophyllene and α-coapene [35].

The fumigant toxicity bioassay on the essential oil of *L. camara* revealed that it can substantially affect the life of all the test insects, and the mortality percentage of test insects can increase with an increase in the dosage. Previous studies support the high fumigant toxicity of *L. camara* essential oil, which can also increase with the dose and duration of exposure [7]. In this study, a 50% mortality of pulse beetle was achieved at a lethal concentration of 6.245 mg/L air after 24 h exposure, which is a significantly greater activity when compared with other works employing various *Callasobruchus* species [18,36]. In a study regarding the fumigant toxicity of essential oils of *Citrus sinensis* and *Mentha pulegium* on red flour beetle and *C. maculatus*, the LC_50_ obtained was much higher than that of this study [37], indicating a greater fumigant activity of *L. camara* essential oil. In a similar study on the fumigant potential of *M. pulegium* against pulse beetle, the LC_50_ obtained was higher compared to that of *L. camara* against the same storage pest [38]. The LC_50_ of *Callistemon sieberi* and *Eucalyptus* sp. essential oil against red flour beetle was also higher when compared to the LC_50_ of *L. camara* essential oil against red flour beetle [39]. An experimental investigation to assess the fumigant toxicity of the β-Caryophyllene was administered against the maize weevil, *Sitophilus zeamais*, resulting in noteworthy lethality upon fumigant toxicity [34]. Another fumigant toxicity evaluation involved essential oil derived from β-Caryophyllene-rich curry leaf essential oils and revealed remarkable fumigant toxicity against *T. castaneum* and *C. maculatus* [40].

*L. camara* essential oil has excellent repellency against test insects, especially red flour beetle. A study on the bioactivity of lantana essential oil against the pulse beetle supports the efficient repellent activity of the essential oil [41]. Many research studies have been conducted on assessing the repellent activity of various other essential oils, including *Mentha piperita*, Rosemary, *Cananga odorata*, and others, against test species such as *T. castaneum*, *L. serricorne*, and *C. chinensis* [41,42], which were found to be equally effective. The repellent effect of lantana essential oil on mosquitoes is also high [20]. The essential oil of lantana possesses various volatile compounds, which can account for its high repellent activities. Numerous research studies into the insecticidal properties of different essential oils, with β-Caryophyllene as the predominant component, have demonstrated noteworthy impacts on storage and other agricultural pests through significant repellent effects [40,43]. Another repellent activity evaluation involved essential oil derived from a Piper species, *Piper guineense,* and revealed remarkable repellency. The major components of the *Piper guineense* essential oil were caryophyllene and α-coapene [35]. The studies showed the significant repellent effects of β-Caryophyllene against stored product insects [41].

This study showed very little phytotoxicity at higher concentrations in terms of plumule and radicle growth and no significant effect on the germination of paddy seeds. Some studies on the phytotoxic effect of *L. camara* revealed that it affects the germination of specific crops like *Amaranathus hybridus* [44]. However, various studies showed little or no significance in phytotoxicity towards seed germination [28,41,45]. The germination of paddy seeds remained consistent across all tested levels, aligning with the prescribed minimum standard of 80% set by Indian regulations [45]. Essential oils from *L. camara* treatment proved non-inhibitory to the germination process, confirming the practical feasibility of employing these oils for controlling stored grain insect pests within storage facilities and godowns.

## 4. Materials and Methods

### 4.1. Collection of Plant Material and Extraction of Essential Oil

*Lantana camara*, commonly regarded as a weed species, was collected from Kerala Agricultural University, Thrissur, India (10.5449° N, 76.2864° E) in August 2023. Leaves of *L. camara* were chopped into small pieces and kept for shade-drying for 7 days. The round bottom flask of a modified Clevenger-type apparatus was fed with dried leaves to carry out hydro distillation at 100 °C for 6 h [41]. Essential oil was obtained as the upper organic phase. Anhydrous sodium sulphate was added to the essential oil to remove its water content.

The following formula was used to determine the lantana oil yield on a fresh-weight basis.

Yield (%*_v_*_/*w*_) = V_E0_ (volume of dry essential oil)/W_l_ (weight of leaf taken) × 100

### 4.2. Essential Oil Chemical Characterization Using GC-MS/MS

The gas chromatography–mass spectrometry (GC-MS) technique was employed to unveil the phytochemical constituents of *L. camara* essential oil [11]. This chemical characterization employed the TSQ 8000 Evo system from Thermo Fisher Scientific (Waltham, MA, USA), featuring an autosampler and a TG-1MS capillary column, with helium as the carrier gas. A split ratio of 1:200 was used for the sample introduction. The acquired mass spectral data were rigorously assessed using the Xcalibur 1.1 software tool, and component identification was achieved by referencing the NIST library, facilitating a comprehensive understanding of the essential oil composition. The temperature steadily increased, and the spectra were scanned from 35 *m*/*z* to 500 *m*/*z*. Thereafter, the peak area of each chemical constituent was calculated to determine the relative percentages.

### 4.3. Test Insect Culture

*Tribolium castaneum* and *L. serricorne* were reared using wheat flour. A plastic bottle measuring 25 cm in height and 12 cm in width was loaded with wheat flour (200 g) containing brewer’s yeast 5% (*w*/*w*) that had been sterilized. The adult insects were introduced into the plastic bottle for an oviposition period of 5 days and were then relocated to new containers after egg laying. The plastic bottles used for rearing were closely monitored to obtain uniformly aged adults. The temperature maintained was 28 ± 2 °C with a relative humidity of 85 ± 5% [41].

Green gram grains were used for the maintenance of *C. chinensis*. To avoid the chance of any insect infestation, the green gram was washed properly and dried using a hot air oven at a temperature of 60 °C. Twenty five *C. chinensis* adults were then placed in a sterile plastic bottle (1.5 L) containing green gram. Adult beetles were transferred into fresh jars after 5 days. The temperature and relative humidity maintained were the same as that for *T. castaneum* and *L. serricorne.*

### 4.4. Insecticidal Activities

#### 4.4.1. Contact Toxicity

The contact toxicity of *L. camara* essential oil was assessed using the residual film technique [28]. Contact toxicity was assessed against three test insects—*T. castaneum*, *L. serricorne*, and *C. chinensis*. A range of concentrations of essential oil solutions with acetone as the solvent was prepared for the assessment. Clean Petri plates of 9 cm diameter were taken, and 1 mL of acetone solution containing essential oil of each concentration was evenly applied to the Petri plates. The Petri plate was slightly swirled to ensure uniform spread. It was kept for drying for 30 min. After drying, 10 adult specimens of each test insect were meticulously introduced into the Petri dish, which was subsequently sealed with its lid. The number of dead insects was noted after 24 and 48 h. LC_50_ and LC_90_ values were found after correcting the mortality by Abbott’s equation [46]. Each treatment was triplicated.

#### 4.4.2. Fumigant Toxicity

In this study, an airtight plastic bottle with a tight cap was used to assess the fumigation toxicity of *L. camara* essential oil [11]. The volume of the plastic bottle taken was 70 mL. The specified amount of pure essential oil was administered onto circular discs made of Whatman No. 1 filter paper using a micropipette. The circular discs were then affixed to the cap of the plastic bottle so that it was suspended inside the bottle without making any contact with the insect. Ten adults of the test insect were transferred into the plastic bottle, which was tightly closed. All treatments were repeated thrice, and a plastic bottle with no oil was used as the control. The mortality of test insects was monitored at 24 h and 48 h. The LC_50_ and LC_90_ values were calculated after correcting for control mortality [20].

#### 4.4.3. Repellent Activity

The research employed a technique known as modified area preference to investigate the repellent properties of *L. camara* essential oil [41]. Circular discs of 9 cm diameter were made out of Whatman No. 1 filter paper and divided into two halves. The two halves were placed in a Petri plate of 9 cm diameter. One half was marked as control, and the other as treatment. Different concentrations of essential oil were prepared with acetone as the solvent. Acetone (0.5 mL) was evenly applied to the control fraction of filter paper, and the treatment half was applied with different concentrations of prepared acetonic leaf essential oil solution. Ten adults of each test insect were introduced to the Petri plates, and the Petri plates were closed with a lid with perforations to avoid fumigant action. The number of test insects in each fraction was monitored at 1st, 2nd, 3rd, 4th, 5th and 6th hour. Each concentration was replicated thrice. Percentage repellency (PR) values were calculated as [47]

Percentage Repellency = [(NC − NT)/(NC + NT)] × 100,

where NC = number of insects in the control area; NT = number of insects in the treated area.

The following classes were used for grouping the percentage repellencies obtained:

Class 0 (0 to 0.1%), Class I (0.2 to 10%), Class II (20.1 to 40%), Class III (40.1 to 60%), Class IV (60.1 to 80%) and Class V (80.1 to 100%).

### 4.5. Phytotoxicity Study on Grains

To look into the possible phytotoxicity effects of essential oil obtained from *L. camara* on seed germination, studies on the phytotoxicity of paddy seeds and seedling growth were carried out [41,45]. For this, 0.01% tween 80 solution was prepared with distilled water. Three different concentrations of lantana leaf oil were then prepared using this tween 80 solution. The concentrations taken in this study were 1000, 750 and 500 μg/mL. A mass of 50 g paddy seeds were soaked in 20 mL of each essential oil solution and kept for 1 h. Sterile Petri plates having double layers of Whatman No. 1 filter paper were taken, and 20 soaked seeds from each concentration were placed in the Petri plates after moistening them. Seeds immersed in a 0.01% Tween 80 solution, without any essential oil, were employed as the control. The trial was triplicated, and the percentage of germination, length of plumule, and radicle were recorded.

### 4.6. Data Analysis

ANOVA and Tukey’s HSD test were used for the statistical analysis of the results obtained from repellency and phytotoxicity studies. These calculations were performed using Grapes version 1.1.0 software, as detailed in the work [48]. The LC_50_ and LC_90_ values for the contact and fumigation bioassays were determined using the Polo Plus 2.0 software.

## 5. Conclusions

As the adverse effects of synthetic chemicals on the environment and human health continue to escalate, it is imperative to seek alternatives that can provide equivalent or superior results to those of synthetic chemicals. With the ongoing advancements in biopesticides, *L. camara* has emerged as a viable and effective option. This study demonstrated that the essential oil of *L. camara* exhibited no appreciable phytotoxicity during paddy seed germination and displayed exceptional contact, fumigant, and repellent toxicities against the targeted insects. *L. camara* possesses various ranges of phytochemicals to confer the same. Treatment with essential oils did not compromise the germination rate, affirming the practical viability of utilizing these oils to combat stored grain insect pests in godowns and food storage facilities. Hence, there is significant potential in harnessing *L. camara* as a biopesticide and a means to utilize a noxious weed that poses persistent challenges to the ecosystem. Hence, essential oil-based biopesticides have the potential to serve as a viable substitute for synthetic pesticides within the realm of sustainable pest management. Moreover, exploring nanoformulations of these essential oils can be a crucial future research endeavour, focusing on ensuring stability and practical applicability in countering stored grain insect pests.

## Figures and Tables

**Table 1:** Chemical constituents of *L. camara* leaf oil.

Peak No.	RT ^a^	Compounds	RSI ^b^	%RA ^c^
1.	5.48	α-Pinene	945	1.13
2.	6.09	α-Phellandrene	919	0.91
3.	6.97	Limonene	892	0.35
4.	7.06	α-Ocimene	917	0.53
5.	7.29	2,7-Dimethyl-2,6-octadien-4-ol	864	0.33
6.	7.43	γ-Terpinene	910	0.32
7.	7.90	(+)-4-Carene	897	0.30
8.	9.39	Terpinen-4-ol	895	0.25
9.	12.63	α-Cubebene	910	0.21
10.	13.21	α-Copaene	911	4.11
11.	13.87	Isocaryophillene	905	0.24
12.	14.21	Caryophyllene	961	69.96
13.	14.33	α-ylangene	848	0.61
14.	14.58	Benzene, 1-(1,5-dimethylhexyl)-4-methyl-	840	0.27
15.	14.87	Humulene	909	0.88
16.	15.30	γ-Muurolene	881	0.24
17.	15.76	Guaia-1(10),11-diene	895	0.91
18.	15.93	Davana ether	786	0.37
19.	16.15	Isoaromadendrene epoxide	815	0.97
20.	16.29	Isoledene	819	12.00
21.	16.97	Aromadendrene oxide-(2)	817	0.42
22.	17.57	Lilac alcohol B, Lilac alcohol D	809	1.80
23.	17.65	Caryophyllene oxide	914	0.73
24.	18.04	Hexanamide, N-(2,6-dimethyl phenyl)-	875	1.37

^a^ Retention time; ^b^ Reverse similarity index; ^c^ Relative area (peak area relative to the total peak area).

**Table 2:** Contact toxicity of *L. camara* essential oil against major storage insect pests at different exposure times.

Insects	Exposure Time (h)	LC_50_ ^a^ (mg/cm^2^)	LC_90_ ^a^ (mg/cm^2^)	Slope ± SEM ^b^	χ^2^ (df)
*T. castaneum*	24	8.93 (8.06–9.81)	13.54 (11.91–17.13)	0.35 ± 0.18	0.40
	48	7.92 (7.21–8.61)	10.47 (9.58–11.90)	1.07 ± 0.22	0.04
*L. serricorne*	24	4.82 (3.91–6.32)	17.47 (11.41–40.31)	0.73 ± 0.23	0.16
	48	3.41 (2.62–4.44)	15.57 (10.01–21.04)	0.91 ± 0.22	0.42
*C. chinensis*	24	1.83 (1.21–2.51)	6.27 (4.59–9.79)	1.77 ± 0.28	0.29
	48	0.45 (0.21–0.68)	5.06 (2.68–8.58)	1.64 ± 1.22	0.62

^a^ Values in the bracket represent lower and upper confidence limit; ^b^ SEM: Mean standard error; χ^2^: chi-square.

**Table 3:** Fumigant toxicity of *L. camara* essential oil against major storage insect pests at different exposure times.

Insects	Exposure Time (h)	LC50 ^a^ (mg/L Air)	LC90 ^a^ (mg/L Air)	Slope ± SEM ^b^	χ^2^ (df)
*T. castaneum*	24	16.70 (15.75–18.08)	23.21 (20.67–29.06)	−10.95 ± 1.87	1.98
	48	14.47 (13.51–15.45)	20.97 (18.85–25.67)	−9.23 ± 1.63	2.44
*L. serricorne*	24	4.14 (3.03–4.98)	10.91 (8.29–20.94)	−1.87 ± 0.52	0.76
	48	2.53 (0.82–3.61)	8.62 (6.53–16.34)	−0.97 ± 0.54	0.04
*C. chinensis*	24	6.24 (4.87–9.43)	27.36 (15.05–128.03)	−1.58 ± 0.32	1.46
	48	3.07 (1.77–5.25)	25.85 (11.11–426.17)	0.58 ± 0.18	3.88

^a^ Values in the bracket represent lower and upper confidence limit; ^b^ SEM: Mean standard error; χ^2^: chi square.

**Table 4:** Repellent activities of *L. camara* essential oil against major storage insect pests at different times of exposure.

Insect	Dosage (mg/cm^2^)	Mean Repellency (%)	Class
*T. castaneum*	1	42.78 ± 9.98 ^a^	III
	2	62.22 ± 6.88 ^a^	IV
	3	63.33 ± 13.82 ^a^	IV
	4	64.44 ± 18.21 ^a^	IV
	5	65.56 ± 21.67 ^a^	IV
*L. serricorne*	1	37.78 ± 18.69 ^a^	II
	2	56.10 ± 33.56 ^a^	III
	3	57.78± 13.77 ^a^	III
	4	62.22 ± 17.72 ^a^	IV
	5	64.44 ± 15.0 ^a^	IV
*C. chinensis*	0.1	38.89 ± 2.72 ^b^	II
	0.2	61.11 ± 20.83 ^ab^	IV
	0.3	61.67 ± 12.95 ^ab^	IV
	0.4	62.22 ± 16.28 ^ab^	IV
	0.5	64.44 ± 13.77 ^a^	IV

^a,b^ Means in the same column that are preceded by the same letter do not differ significantly (*p* < 0.05).

**Table 5:** Germination % of rice seeds when treated with *L. camara* essential oil at different times of exposure.

	Seed Germination (%) of Treatments after			
Concentration (µg/mL)	48 h	72 h	96 h	120 h
500	90.0 ± 5.74 ^a^	96.67 ± 0.0 ^a^	100.0 ± 0.0 ^a^	100.0 ± 0.0 ^a^
750	90.0 ± 10.0 ^a^	93.33 ± 5.74 ^a^	96.67 ± 5.74 ^a^	96.78 ± 5.74 ^a^
1000	96.67 ± 5.74 ^a^	96.67.0 ± 0.0 ^a^	100.0 ± 0.0 ^a^	100.0 ± 0.0 ^a^
^b^ Control	93.33 ± 5.74 ^a^	96.67 ± 5.74 ^a^	96.67 ± 5.74 ^a^	96.67 ± 5.74 ^a^
F value	0.61	1.83	1.58	0.93
*p* value	0.62	0.21	0.24	0.12

^a^ Means in the same column that are preceded by the same letter do not differ significantly (*p* < 0.05); ^b^ Distilled water.

## Data Availability

The data presented in this study are available on request from the corresponding author. The data are not publicly available due to ongoing research related to this work.

## References

[1] Qin Z., Zhang J.E., DiTommaso A., Wang R.L., Liang K.M. (2016). Predicting the Potential Distribution of *Lantana camara* L. Under RCP Scenarios Using ISI-MIP Models. Clim. Change.

[2] Shackleton R.T., Witt A.B.R., Nunda W., Richardson D.M. (2016). *Chromolaena odorata* (Siam Weed) in Eastern Africa: Distribution and Socio-Ecological Impacts. Biol. Invasions.

[3] Negi G.C.S., Sharma S., Vishvakarma S.C.R., Samant S.S., Maikhuri R.K., Prasad R.C., Palni L.M.S. (2019). Ecology and Use of *Lantana camara* in India. Bot. Rev..

[4] Priyanka N., Joshi P. (2013). A Review of *Lantana camara* Studies in India. Int. J. Sci. Res. Publ..

[5] Shankar U., Abrol D.P. (2012). Integrated Pest Management in Stored Grains.

[6] Satya S., Kadian N., Kaushik G., Sharma U. Impact of Chemical Pesticides for Stored Grain Protection on Environment and Human Health. https://www.semanticscholar.org/paper/Impact-of-chemical-pesticides-for-stored-grain-on-Satya-Kadian/846312a3e0a1055a0b6bb77fd2c387a24bd0e62b.

[7] Zoubiri S., Baaliouamer A. (2012). Chemical Composition and Insecticidal Properties of *Lantana camara* L. Leaf Essential Oils from Algeria. J. Essent. Oil Res..

[8] Narayanankutty A., Visakh N.U., Sasidharan A., Pathrose B., Olatunji O.J., Al-Ansari A., Alfarhan A., Ramesh V. (2022). Chemical Composition, Antioxidant, Anti-Bacterial, and Anti-Cancer Activities of Essential Oils Extracted from Citrus Limetta Risso Peel Waste Remains after Commercial Use. Molecules.

[9] Albaqami J.J., Hamdi H., Narayanankutty A., Visakh N.U., Sasidharan A., Kuttithodi A.M., Famurewa A.C., Pathrose B. (2022). Chemical Composition and Biological Activities of the Leaf Essential Oils of *Curcuma longa*, *Curcuma aromatica* and *Curcuma angustifolia*. Antibiotics.

[10] Kuttithodi A.M., Narayanankutty A., Visakh N.U., Job J.T., Pathrose B., Olatunji O.J., Alfarhan A., Ramesh V. (2023). Chemical Composition of the Cinnamomum Malabatrum Leaf Essential Oil and Analysis of Its Antioxidant, Enzyme Inhibitory and Antibacterial Activities. Antibiotics.

[11] Visakh N.U., Pathrose B., Narayanankutty A., Alfarhan A., Ramesh V. (2022). Utilization of Pomelo (*Citrus maxima*) Peel Waste into Bioactive Essential Oils: Chemical Composition and Insecticidal Properties. Insects.

[12] Hanif M.A., Nisar S., Khan G.S., Mushtaq Z., Zubair M. (2019). Essential Oils. Essential Oil Research.

[13] Campolo O., Giunti G., Russo A., Palmeri V., Zappalà L. (2018). Essential Oils in Stored Product Insect Pest Control. J. Food Qual..

[14] Campbell J.F., Athanassiou C.G., Hagstrum D.W., Zhu K.Y. (2022). *Tribolium castaneum*: A Model Insect for Fundamental and Applied Research. Annu. Rev. Entomol..

[15] Edde P.A. (2019). Biology, Ecology, and Control of *Lasioderma serricorne* (F.) (Coleoptera: Anobiidae): A Review. J. Econ. Entomol..

[16] Ramazeame L., Adiroubane D., Govindan K., Jagatheeswari J. Management of Pulse Beetle, *Callosobruchus chinensis* Linn. Using Botanicals. https://www.entomoljournal.com/archives/2014/vol2issue4/PartG/34.pdf.

[17] Sousa E.O., Costa J.G.M. (2012). Genus *Lantana*: Chemical Aspects and Biological Activities. Rev. Bras. Farmacogn..

[18] Zandi-Sohani N., Hojjati M., Carbonell-Barrachina Á.A. (2012). Bioactivity of *Lantana camara* L. Essential Oil against *Callosobruchus maculatus* (Fabricius). Chil. J. Agric. Res..

[19] Bouda H., Tapondjou L.A., Fontem D.A., Gumedzoe M.Y.D. (2001). Effect of Essential Oils from Leaves of Ageratum Conyzoides, *Lantana camara* and *Chromolaena odorata* on the Mortality of *Sitophilus zeamais* (Coleoptera, Curculionidae). J. Stored Prod. Res..

[20] Sharma M., Alexander A., Saraf S., Saraf S., Vishwakarma U.K., Nakhate K.T. (2021). Ajazuddin Mosquito Repellent and Larvicidal Perspectives of Weeds *Lantana camara* L. and *Ocimum gratissimum* L. Found in Central India. Biocatal. Agric. Biotechnol..

[21] Misra L., Saikia A.K. (2011). Chemotypic Variation in Indian *Lantana camara* Essential Oil. J. Essent. Oil Res..

[22] Barros L.M., Duarte A.E., Morais-Braga M.F.B., Waczuk E.P., Vega C., Leite N.F., De Menezes I.R.A., Coutinho H.D.M., Rocha J.B.T., Kamdem J.P. (2016). Chemical Characterization and Trypanocidal, Leishmanicidal and Cytotoxicity Potential of *Lantana camara* L. (Verbenaceae) Essential Oil. Molecules.

[23] Khan M., Mahmood A., Alkhathlan H.Z. (2016). Characterization of Leaves and Flowers Volatile Constituents of *Lantana camara* Growing in Central Region of Saudi Arabia. Arab. J. Chem..

[24] Mora F., Rojas L., Díaz T., Velasco J., Yánez C., Rios N., Carmona J., Pasquale S.A., Nurby Ríos Tesch (2011). Chemical Composition and Antibacterial Activity of the Essential Oil of *Lantana camara* Var. Moritziana. Nat. Prod. Commun..

[25] Oyedeji O.A., Ekundayo O., König W.A. (2003). Volatile Leaf Oil Constituents of *Lantana camara* L. from Nigeria. Flavour Fragr. J..

[26] Liambila N.R., Wesonga J.M., Ngamau C.N., Waudo W. (2021). Pesticidal properties of essential oils of *Lantana camara* L.. African J. Hortic. Sci..

[27] de Sena Filho J.G., Rabbani A.R.C., dos Santos Silva T.R., da Silva A.V.C., Souza I.A., Santos M.J.B.A., de Jesus J.R., de Lima Nogueira P.C., Duringer J.M. (2012). Chemical and Molecular Characterization of Fifteen Species from the *Lantana* (Verbenaceae) Genus. Biochem. Syst. Ecol..

[28] Visakh N.U., Pathrose B., Chellappan M., Ranjith M.T., Sindhu P.V., Mathew D. (2023). Extraction and Chemical Characterisation of Agro-Waste from Turmeric Leaves as a Source of Bioactive Essential Oils with Insecticidal and Antioxidant Activities. Waste Manag..

[29] Rajashekar Y., Ravindra K.V., Bakthavatsalam N. (2012). Leaves of *Lantana camara* Linn. (Verbenaceae) as a Potential Insecticide for the Management of Three Species of Stored Grain Insect Pests. J. Food Sci. Technol..

[30] Rajashekar Y., Raghavendra A., Bakthavatsalam N. (2014). *Acetylcholinesterase* Inhibition by Biofumigant (Coumaran) from Leaves of *Lantana camara* in Stored Grain and Household Insect Pests. BioMed Res. Int..

[31] Du S.-S., Yang K., Wang C., You C., Geng Z., Guo S.-S., Deng Z., Liu Z. (2014). Chemical Constituents and Activities of the Essential Oil from *Myristica fragrans* against Cigarette Beetle *Lasioderma serricorne*. Chem. Biodivers..

[32] Murugesan S., Senthilkumar N., Babu D., Rajasugunasekar D.R. (2016). Chemical Constituents and Toxicity Assessment of the Leaf Oil of *Lantana camara* Linn from Tamilnadu Regions. Asian J. Plant Sci. Res..

[33] Liambila R., Wesonga J., Ngamau C., Waudo W. (2021). Chemical Composition and Bioactivity of *Lantana camara* L. Essential Oils from Diverse Climatic Zones of Kenya against Leaf Miner (*Tuta absoluta* Meyrick). Afr. J. Agric. Res..

[34] Chaubey M. (2022). Terpenes in Maize Weevil Management Insecticidal Property of Terpenes against Maize Weevil, Sitophilus Zeamais (Motschulsky). J. Biopestic..

[35] Esther O.O., Robyn M., Kim P.T., Nelson N.N. (2015). Essential Oil Composition of Different Fractions of Piper Guineense Schumach. Et Thonn from Cameroon Using Gas Chromatography-Mass Spectrometry and Their Insecticidal Effect on *Sitophilus oryzae* (L.). Afr. J. Biotechnol..

[36] Mahmoudvand M., Abbasipour H., Basij M., Hossein Hosseinpour M., Rastegar F., Bagher Nasiri M. (2011). Fumigant Toxicity of Some Essential Oils on Adults of Some Stored-Product Pests. Chil. J. Agric. Res..

[37] Salem N., Bachrouch O., Sriti J., Msaada K., Khammassi S., Hammami M., Selmi S., Boushih E., Koorani S., Abderraba M. (2017). Fumigant and Repellent Potentials of *Ricinus communis* and *Mentha pulegium* Essential Oils against *Tribolium castaneum Lasioderma serricorne*. Int. J. Food Prop..

[38] Lee B.-H., Annis P.C., Tumaalii F., Choi W.-S. (2004). Fumigant toxicity of essential oils from the Myrtaceae family and 1,8-cineole against 3 major stored-grain insects. J. Stored Prod. Res..

[39] Lee B.-H., Lee S.-E., Annis P.C., Pratt S.J., Park B.-S., Tumaalii F. (2002). Fumigant Toxicity of Essential Oils and Monoterpenes against the Red Flour Beetle, *Tribolium castaneum* Herbst. J. Asia-Pac. Entomol..

[40] Visakh N.U., Pathrose B., Narayanankutty A. (2023). Characterization of Secondary Metabolites from the Leaves of Curry Leaf (*Murraya koenigii* L.) Essential Oils with Insecticidal Activities against Stored Product Insects. Biocatal. Agric. Biotechnol..

[41] Visakh N.U., Pathrose B., Chellappan M., Ranjith M.T., Sindhu P.V., Mathew D. (2022). Chemical Characterisation, Insecticidal and Antioxidant Activities of Essential Oils from *Four citrus* spp. Fruit Peel Waste. Food Biosci..

[42] Caballero-Gallardo K., Olivero-Verbel J., Stashenko E.E. (2011). Repellent Activity of Essential Oils and Some of Their Individual Constituents against *Tribolium castaneum* Herbst. J. Agric. Food Chem..

[43] Ma S., Jia R., Guo M., Qin K., Zhang L. (2020). Insecticidal Activity of Essential Oil from *Cephalotaxus sinensis* and Its Main Components against Various Agricultural Pests. Ind. Crops Prod..

[44] Verdeguer M., Blázquez M.A., Boira H. (2009). Phytotoxic Effects of *Lantana camara*, *Eucalyptus camaldulensis* and *Eriocephalus africanus* Essential Oils in Weeds of Mediterranean Summer Crops. Biochem. Syst. Ecol..

[45] Jaya, Singh P., Prakash B., Dubey N.K. (2012). Insecticidal Activity of *Ageratum conyzoides* L., *Coleus aromaticus* Benth. and *Hyptis suaveolens* (L.) Poit Essential Oils as Fumigant against Storage Grain Insect *Tribolium castaneum* Herbst. J. Food Sci. Technol..

[46] Abbott W.S. (1925). A Method of Computing the Effectiveness of an Insecticide. J. Econ. Entomol..

[47] Debbabi H., El Mokni R., Nardoni S., Chaieb I., Maggi F., Nzekoue F.K., Caprioli G., Hammami S. (2021). Chemical Diversity and Biological Activities of Essential Oils from Native Populations of *Clinopodium menthifolium* Subsp.. Ascendens (Jord.) Govaerts. Environ. Sci. Pollut. Res. Int..

[48] Gopinath P., Parsad R., Joseph B., Adarsh V.S. (2021). GrapesAgri1: Collection of Shiny Apps for Data Analysis in Agriculture. J. Open Source Softw..

